# COMT and MTHFR Genetic Variants Combined Effects on Adolescent Idiopathic Scoliosis Progression

**DOI:** 10.7150/jgen.104110

**Published:** 2025-01-01

**Authors:** Jessica Wright, Adrijana Kekic, Ann Vincent, Jana Kay Lacanlale, Razan El Melik, Eric Matey, Mark Morningstar

**Affiliations:** 1 Mayo Clinic, Rochester, MN, USA.; 2 Mayo Clinic, Phoenix, AZ, USA.; 3 Natural Wellness & Pain Relief Center, Grand Blanc, MI, USA.

## Abstract

**Purpose:** Genetic variants encoding both low COMT and MTHFR activity are associated with idiopathic scoliosis. The combined impact of *COMT* and* MTHFR* on progression of adolescent idiopathic scoliosis (AIS) is unknown. This study investigated if *COMT* and *MTHFR* low activity variants are associated with AIS progression.

**Methods:** Patients with AIS, at least two Cobb angle measurements in adolescence, and those with both low *COMT* (rs4680 AA) and low *MTHFR* (A1298C AC and C677T CT; A1298C AA and C677T TT) activity (Group 1) or those with intermediate or high *COMT* (rs4680 AG or GG) and *MTHFR* (A1298C AA and C677T CT; A1298C AC and C677T CC; A1298C AA and C677T CC) activity (Group 2) were included. Those with neuromuscular or syndromic scoliosis were excluded. The primary outcome was progression of scoliosis, defined as a Cobb angle increase of at least 20 degrees or spinal surgery between the time of diagnosis and skeletal maturity. The primary outcome was analyzed via a Chi-square test.

**Results:** Seventy-two patients with AIS diagnosis and required Cobb angle measurements had both *COMT* and *MTHFR* results that met criteria for Group 1 (n=41) or Group 2 (n=31). Regarding the primary outcome, 78.0% (32/41) in Group 1 progressed versus 48.4% (15/31) of patients in Group 2 (p=0.009).

**Conclusion:** Significantly more patients with both low *COMT* and low *MTHFR* activity variants had progression of AIS than those with intermediate or normal activity variants of *COMT* and *MTHFR*. Further understanding the role of *COMT* and *MTHFR* may inform research regarding treatment modalities.

## Introduction

Adolescent idiopathic scoliosis (AIS) is the most common type of idiopathic scoliosis in children. The etiology of AIS is multifactorial. Genetic variants involving extracellular matrix proteins, abnormalities in spine biomechanics, neurotransmitters such as catecholamines, hormonal factors such as sex hormones and melatonin, and environmental and lifestyle factors have been reported to be among the many contributors involved in AIS initiation or progression^1^. Females have an increased incidence and progression risk compared with males^2^. In girls with AIS, delayed onset of menarche is more common^3^. These sex differences and the influence of menarche timing have prompted hypotheses that differences in endocrine hormones such as estrogen may play a role in AIS susceptibility.

There is abundant literature regarding the role of estrogen in collagen production and maintenance of a healthy bone density^4^. AIS is associated with low bone density and part of this may relate to abnormal estrogen receptor expression, signaling pathways or metabolism^5^-^7^. A variant of *COL11A1*, the gene encoding one of the proteins of collagen is associated with AIS; expression of *COL11A1* has been reported to have associations with levels of estrogen receptors, further indicating the potential role of estrogen in AIS. Catechol-O-methyltransferase (COMT), an enzyme encoded by the *COMT* gene, influences estrogen metabolism by inactivating and detoxifying catecholestrogens formed by conversion of estrone and estradiol to catechol estrogen 4-hydroxyestrogen. *COMT* rs4680 (Val158Met) AA (Met/Met) genotype is associated with 3-4 fold reduced COMT function than the GG (Val/Val) genotype. Morningstar and colleagues have reported that more patients with scoliosis harbored the *COMT* rs4680 AA genotype than controls. Therefore, both reduced COMT function and abnormalities in estrogen levels related to *COMT* variants could be important etiologies in AIS. Thus, factors that influence estrogen signaling and genetic variants such as *COL11A1* and *COMT* require further study.

Similarly, melatonin deficiency, abnormalities in melatonin signaling, and alterations of the gene encoding methylenetetrahydrofolate reductase (MTHFR), specifically reduced function variants of the gene, have been implicated in AIS etiology. MTHFR is an enzyme involved in converting folic acid to reduced folate and is one of several important enzymes in the production of melatonin^8^-^11^. A retrospective study demonstrated the relationship between MTHFR variants and incidence of idiopathic scoliosis. In this study, 23 of 44 patients with a history of idiopathic scoliosis had a positive MTHFR variant (defined as homozygous A1298C, homozygous C677T, or heterozygous for both C677T and A1298C) versus 11 of the 44 patients in the control group (P < 0.01)^12^. In a case report, an infant with severe scoliosis was noted to have two severely reduced function variants in the MTHFR gene, resulting in severe MTHFR deficiency^13^. In this infant, the degree of spinal curvature reportedly improved after four months of a medication regimen consisting of folate, cyanocobalamin, pyridoxine, acetylsalicylic acid, and betaine, supporting the role of MTHFR and folate in the etiology of scoliosis^13^. In another prospective study that measured melatonin levels in patients with AIS, the authors reported that more patients with lower melatonin levels had disease progression compared with those with normal levels^14^. Although the reasons for low melatonin were not described in the study, it may be related to MTHFR abnormalities, further strengthening the need to examine the role of MTHFR variants in AIS.

*COMT* and *MTHFR* have not been extensively studied in AIS and large knowledge gaps remain. The aim of this study is to determine if variants associated with low COMT and MTHFR activity are associated with AIS progression.

## Methods

This retrospective cohort study was deemed exempt by Mayo Clinic Institutional Review Board. Inclusion criteria were diagnosis of AIS, presence of a Cobb angle measurement at diagnosis and a second measurement at skeletal maturity, and one of the two following genetic variant combinations: both low COMT and MTHFR activity as defined below for Group 1 or intermediate or high activity of both COMT and MTHFR as defined below for Group 2. Two predetermined groups for analysis were as follows: Group 1 had both low COMT activity (rs4680 AA genotype) and low MTHFR activity, which was defined as less than 50% activity^15^ by the following genotype combinations: heterozygous for both C677T and A1298C; homozygous for the C677T variant and A1298C wild type. Group 2 had intermediate or high COMT activity (rs4680 AG or GG genotypes) and not low MTHFR activity, defined as the following genotype combinations: A1298C wild type and heterozygous C677T; A1298C heterozygous and C677T wild type and; A1298C and C677T wild type. Individuals with a diagnosis of that would characterize the scoliosis as congenital, neuromuscular or syndromic were excluded. Additionally, patients with *COMT* and *MTHFR* variant combinations that did not fit into criteria for Group 1 nor Group 2 were excluded.

*COMT* and *MTHFR* were tested by OneOme, LLC and AssureRx® within the cohort of 5 patients from the Mayo Clinic site. DNA derived from saliva samples were tested by 23andMe® which also contained the same *COMT* and *MTHFR* variants tested by OneOme. The raw data from 23andMe® was used by Functional Genomic Analysis^TM^ software to interpret *COMT* and *MTHFR* test results for 67 patients from the Natural Wellness & Pain Relief Center site.

The primary outcome was significant progression of scoliosis between initial diagnosis and skeletal maturity or spinal surgery to correct the scoliosis curve, whichever came first. Progression was defined for the purpose of this study as an increase in the Cobb angle of at least 20 degrees or requiring spinal surgery between the time of diagnosis and skeletal maturity. Group 1 and Group 2 outcomes of progression were analyzed via a Chi-square test using GraphPad Software^©^, Boston, MA.

## Results

A total of 102 subjects with an AIS diagnosis and required Cobb angle measurements had both *COMT* and *MTHFR* testing. Forty one (40.2%) of these patients had both low COMT (rs4860 AA) and MTHFR activity (Group 1), 30.4% (31/102) had intermediate or normal activity of both genes (Group 2), and 29.4% (30/102) were excluded from analysis due to low activity for either genes but not both. Therefore, a total of 72 patients met inclusion criteria for this study. A majority of patients were females with the same proportion of females in Group 1 as Group 2, 29/41 (70.7%) and 22/31 (70.7%), respectively. All patients were Caucasian.

With regard to the primary outcome, 78.0% (32/41) in Group 1 had progression of 20 degrees or greater versus 48.4% (15/31) of patients in Group 2 (p=0.009). The average age at diagnosis for Group 1 was 11.3 years and 11.8 years for Group 2. The largest Cobb angle in either group at skeletal maturity or prior to spinal fusion surgery was 68 degrees.

## Discussion

To our knowledge, this is the first study to report an association between the combination of *MTHFR* and *COMT* variants and curve progression in patients with AIS. Although two previous studies reported an association of *COMT* and *MTHFR* individually with AIS incidence, no previous study has examined both genes in the context of disease progression^1^,^12^. Etiology of AIS is multifactorial, and further studies have improved our understanding of this disease as multifaceted with a polygenetic background^3^. Our study is significant because at the current time, there are no genetic prognostication tools available for this condition and genetic testing may be a potential avenue for future research to identify individualized treatment according to genetic phenotypes.

Similar to our study, two other separate studies reported an association between low activity variants of *COMT* and *MTHFR* and idiopathic scoliosis, respectively^12^. However, these studies examined the relationship between these genes and incidence of the disease but not disease progression. Similar to multiple other studies showing AIS predominance in females, our cohort also demonstrated that a majority of patients in the cohort were female. Our findings demonstrate that the combination of both low COMT and MTHFR activity further supports the theory of the relationship between AIS and factors influencing estrogen metabolism such as *COMT*. While a single genetic variant alone may not be enough to result in AIS progression, perhaps multiple genetic variants combined have a greater effect.

Current AIS management strategies are not sufficient to prevent progression in all patients. Management of AIS during adolescence prior to skeletal maturity generally consists of bracing for patients meeting specific bracing criteria such as curves 25-45 degrees, scoliosis-specific exercises, and surgical management for severe curves^3^. Progression still occurs despite bracing and scoliosis-specific exercises. With bracing for 18 hours per day, 72% of adolescent subjects had treatment success, defined by a single study as lack of curve progression beyond 50 degrees at skeletal maturity, which leaves 28% of patients with substantial progression^16^. When a group of exercises known as the Schroth method was added to standard of care for adolescents with AIS, Cobb angles were reduced with measures of quality of life increased, although the Cobb angle reduction was not beyond the threshold for a clinically important difference^17^. Long-term sequelae of untreated or suboptimally treated AIS are curve progression, back pain, cardiopulmonary morbidity, and psychosocial issues^3^. In skeletally mature patients, curves less than 30 degrees are generally not expected to progress^3^. However, curves of 30-50 degrees at skeletal maturity were reported to progress 10 to 15 degrees during one's lifetime^3^. Curves greater than 50 degrees can progress by 1 degree per year^3^. There is an opportunity to leverage personalized medicine in such a heterogeneous disease. By targeting potential biomarkers associated with AIS etiology such as *COMT* and *MTHFR*, therapeutic modalities targeted specifically to these biomarkers could be further studied with the goal of decreasing the risk of progression in the adolescent years before skeletal maturity as this is when progression risk is the greatest^18^.

COMT is involved in other metabolic activities besides estrogen metabolism which could introduce an alternative explanation to our findings. COMT has been shown to play a role in pain perception by regulating epinephrine and norepinephrine levels. Low COMT activity is associated with susceptibility to nociceptive pain due to high adrenergic signaling whereas high COMT activity is associated with susceptibility to neuropathic pain^19^. *COMT* rs4680 AA genotype is associated with an increased risk in chronic pain syndromes including fibromyalgia^20^. In both healthy volunteers and patients who underwent shoulder surgery, those with the AA genotype also reported the most intense pain^21^. The opposite effect is seen with high COMT activity, which is associated with lower sensitivity to pain after hip surgery^21^. Therefore, it is possible that there was a selection bias in our cohort for patients seeking chiropractic and medical care in combination with genetic testing that included *COMT* and *MTHFR* genes due to increased pain perception.

If *MTHFR* and *COMT* associations can be confirmed with larger studies, folate administration, which bypasses the deficiency of folic acid conversion to folate, could be a potential next step for clinical research to determine if folate supplementation may reduce AIS progression. Similar to our findings of MTHFR and COMT risk factors involved in AIS progression, several other studies suggest that melatonin deficiency also affects the prognosis of idiopathic scoliosis (IS), as previously stated. In one small prospective study, melatonin supplementation resulted in numerically lower rates of progression in those with low melatonin levels compared with those who had low levels and no supplementation^14^. Confirmatory studies are needed to determine if melatonin supplementation decreases AIS progression. Investigating a targeted therapy specific to the AIS etiology and prognosis is an important step in advancing prevention and management strategies for AIS.

Understanding factors that influence curve behavior is critical to prognostication and developing potential management strategies to prevent curve progression in both adolescence and adulthood. Factors identified related to progression include magnitude of the curve, age at diagnosis, Risser sign, and menarche status^22^-^24^. For long term prognostication, Tan *et al.* found that an initial Cobb angle of 25 degrees or higher was an important predictor for a curve of 30 degrees or more at skeletal maturity^25^.

Other factors implicated in the rapid progression of AIS are *SOX9* and *KCNJ2* variants, Lenke type 4 curves associated with gene loci 6q24 and 10q24, microRNA 4300 genes, increased calmodulin levels, decreased melatonin levels, and a positive ScoliScore^26^. *FBN1*, *FBN2*, and variants near *SOX9*, and *KCNJ2* are associated with severe scoliosis (greater than 40 degrees). Variants in genes coding for calmodulin 1 (*CALM1* rs12885713), estrogen receptor 1 (*ER1* rs2234693) was associated with progressive AIS with severe spine deformity (Cobb angle > 40 degrees and different curve patterns). Tryptophan hydroxylase (*TPH1* rs10488682), insulin-like growth factor (*IGF1* rs5742612), neurotrophin 3 (*NTF3* gene promoter at rs11063714), interleukin-17 receptor C (*IL17RC* rs708567), melatonin receptor 1B (*MTNR1B* rs4753426) were identified as risk factors for scoliosis curve progression^27^. The aforementioned genetic variants were unknown in our cohort; therefore, it is unknown if these genes, if present in our cohort, had any influence on our findings.

## Conclusion

The combination of both low activity of MTHFR and COMT was found to be a set of genetic risk factors associated with progression of AIS in adolescent years between initial diagnosis and skeletal maturity. Understanding potential underlying genetic risk factors for AIS progression may support future studies examining potential prevention or treatment strategies to augment important mainstays of treatment such as bracing or scoliosis-specific exercises in select individuals. If further studies can confirm the association, we found with the combination of MTHFR and COMT low activity variants, this body of evidence could be used to support investigation of potential therapeutic approaches such as folate and melatonin supplementation or other modalities targeting the MTHFR and COMT pathways to reduce the risk of AIS progression.

## Abbreviations

AIS: adolescent idiopathic scoliosis
COMT: catechol-o-methyltransferase
MTHFR: methylenetetrahydrofolate reductase

## References

[1] Mark W (2018). Morningstar Megan N Strauchman, Clayton J. Stitzel, Brian Dovorany, Aatif Siddiqui. Catechol-O-Methyl Transferase (COMT) Enzyme Gene Variations in Patients with Idiopathic Scoliosis: A Clinical Chart Review. Open Journal of Genetics.

[2] Lonstein JE (2006). Scoliosis: surgical versus nonsurgical treatment. Clin Orthop Relat Res.

[3] Addai D, Zarkos J, Bowey AJ (2020). Current concepts in the diagnosis and management of adolescent idiopathic scoliosis. Childs Nerv Syst.

[4] Brown MA, Haughton MA, Grant SF, Gunnell AS, Henderson NK, Eisman JA (2001). Genetic control of bone density and turnover: role of the collagen 1alpha1, estrogen receptor, and vitamin D receptor genes. J Bone Miner Res.

[5] Yu WS, Chan KY, Yu FW, Yeung HY, Ng BK, Lee KM (2013). Abnormal bone quality versus low bone mineral density in adolescent idiopathic scoliosis: a case-control study with in vivo high-resolution peripheral quantitative computed tomography. Spine J.

[6] Zhou C, Wang H, Zou Y, Fang H (2015). [Research Progress of Role of Estrogen and Estrogen Receptor on Onset and Progression of Adolescent Idiopathic Scoliosis]. Zhongguo Xiu Fu Chong Jian Wai Ke Za Zhi.

[7] Kotwicki T, Tomaszewski M, Andrusiewicz M, Sliwa A, Rusin B, Kotwicka M (2022). Estrogen Receptor Type 1 and Type 2 Presence in Paravertebral Skeletal Muscles: Expression Level and Relation to Phenotype in Children with Idiopathic Scoliosis. Genes (Basel).

[8] Vidmar Golja M, Smid A, Karas Kuzelicki N, Trontelj J, Gersak K, Mlinaric-Rascan I (2020). Folate Insufficiency Due to MTHFR Deficiency Is Bypassed by 5-Methyltetrahydrofolate. J Clin Med.

[9] Bhatia P, Singh N (2015). Homocysteine excess: delineating the possible mechanism of neurotoxicity and depression. Fundam Clin Pharmacol.

[10] Roman GC, Mancera-Paez O, Bernal C (2019). Epigenetic Factors in Late-Onset Alzheimer's Disease: MTHFR and CTH Gene Polymorphisms, Metabolic Transsulfuration and Methylation Pathways, and B Vitamins. Int J Mol Sci.

[11] Kapoor V, Watson NF, Ball L (2022). Chronic insomnia in the setting of MTHFR polymorphism. J Clin Sleep Med.

[12] Mark W (2017). Morningstar MNS, Clayton J. Stitzel, Brian Dovorany, Aatif Siddiqui. Methylenetetrahydrofolate Reductase (MTHFR) Gene Mutations in Patients with Idiopathic Scoliosis: A Clinical Chart Review. Open Journal of Genetics.

[13] Munoz T, Patel J, Badilla-Porras R, Kronick J, Mercimek-Mahmutoglu S (2015). Severe scoliosis in a patient with severe methylenetetrahydrofolate reductase deficiency. Brain Dev.

[14] Machida M, Dubousset J, Yamada T, Kimura J (2009). Serum melatonin levels in adolescent idiopathic scoliosis prediction and prevention for curve progression-a prospective study. J Pineal Res.

[15] Chango A, Boisson F, Barbe F, Quilliot D, Droesch S, Pfister M (2000). The effect of 677C->T and 1298A->C mutations on plasma homocysteine and 5,10-methylenetetrahydrofolate reductase activity in healthy subjects. Br J Nutr.

[16] Weinstein SL, Dolan LA, Wright JG, Dobbs MB (2013). Effects of bracing in adolescents with idiopathic scoliosis. N Engl J Med.

[17] Schreiber S, Parent EC, Hill DL, Hedden DM, Moreau MJ, Southon SC (2019). Patients with adolescent idiopathic scoliosis perceive positive improvements regardless of change in the Cobb angle - Results from a randomized controlled trial comparing a 6-month Schroth intervention added to standard care and standard care alone. SOSORT 2018 Award winner. BMC Musculoskelet Disord.

[18] Schlosser TP, Vincken KL, Rogers K, Castelein RM, Shah SA (2015). Natural sagittal spino-pelvic alignment in boys and girls before, at and after the adolescent growth spurt. Eur Spine J.

[19] Segall SK, Maixner W, Belfer I, Wiltshire T, Seltzer Z, Diatchenko L (2012). Janus molecule I: dichotomous effects of COMT in neuropathic vs nociceptive pain modalities. CNS Neurol Disord Drug Targets.

[20] Tammimaki A, Mannisto PT (2012). Catechol-O-methyltransferase gene polymorphism and chronic human pain: a systematic review and meta-analysis. Pharmacogenet Genomics.

[21] Machoy-Mokrzyńska A, Starzyńska-Sadura Z, Dziedziejko V, Safranow K, Kurzawski M, Leźnicka K (2019). Association of COMT gene variability with pain intensity in patients after total hip replacement. Scandinavian Journal of Clinical and Laboratory Investigation.

[22] Lonstein JE, Carlson JM (1984). The prediction of curve progression in untreated idiopathic scoliosis during growth. J Bone Joint Surg Am.

[23] Soucacos PN, Zacharis K, Gelalis J, Soultanis K, Kalos N, Beris A (1998). Assessment of curve progression in idiopathic scoliosis. Eur Spine J.

[24] Charles YP, Daures JP, de Rosa V, Dimeglio A (2006). Progression risk of idiopathic juvenile scoliosis during pubertal growth. Spine (Phila Pa 1976).

[25] Tan KJ, Moe MM, Vaithinathan R, Wong HK (2009). Curve progression in idiopathic scoliosis: follow-up study to skeletal maturity. Spine (Phila Pa 1976).

[26] Marya S, Tambe AD, Millner PA, Tsirikos AI (2022). Adolescent idiopathic scoliosis: a review of aetiological theories of a multifactorial disease. Bone Joint J.

[27] Noshchenko A, Hoffecker L, Lindley EM, Burger EL, Cain CM, Patel VV (2015). Predictors of spine deformity progression in adolescent idiopathic scoliosis: A systematic review with meta-analysis. World J Orthop.

